# Does CB-1 in hepatic stellate cells contribute to liver fibrosis? 

**DOI:** 10.1172/JCI155413

**Published:** 2022-01-04

**Authors:** Sophie Lotersztajn, Ariane Mallat

**Affiliations:** Université de Paris, INSERM, U1149, Centre de Recherche sur l’Inflammation (CRI), Laboratoire d’Excellence Inflamex VF-75018, Paris, France.

**Keywords:** Hepatology, Fibrosis

## To the Editor:

The development of drugs targeting the peripheral cannabinoid receptor 1 (CB-1) has been identified as a major therapeutic opening for the management of nonalcoholic fatty liver disease (NAFLD) and liver fibrosis (1). Studies from our laboratory and that of George Kunos identified the endocannabinoid system and CB-1 in hepatic stellate cells (HSCs) and hepatocytes as major drivers of fibrogenesis and steatogenesis, findings that were confirmed by several teams (1, 2). The article by Wang et al. (3) challenges this current view, but we have concerns regarding their data and conclusions.

Wang et al. claim that CB-1 was not induced in hepatocytes or HSCs in their NAFLD and fibrosis models. However, their study was limited to the analysis of CB-1 mRNA variations, and their results contradict a large number of studies involving rodent and human liver samples as well as isolated hepatocytes and HSCs that combined mRNA and protein analysis and functional studies (1, 2). Moreover, we have several methodological concerns: (a) single-cell RNA-Seq was performed in HSCs from high-fat diet–fed mice, a model in which HSCs are hardly activated; (b) the lack of detection of CB-1 in HSCs in human liver samples is questionable, since their histological images did not show fibrosis or markers of HSC activation.

Finally, the authors used mice deficient for CB-1 in HSCs to evaluate the role of these cells in fibrogenesis and found no differences in the amount of fibrosis between KO and wild-type mice. On the basis of these findings, they questioned the antifibrogenic potential of peripherally restricted CB-1 antagonists. However, they did not perform functional studies to validate the impact of CB-1 deletion in HSCs on CB-1 signaling and/or fibrogenic properties. Moreover, Picrosirius red staining quantification of fibrosis was performed on a limited number (*n* = 3) of animals per group. Finally, we believe that their conclusion is simplistic, since liver fibrosis results from coordinated interactions of HSCs with macrophages, endothelial cells, and hepatocytes, all of which express CB-1. In fact, the antifibrogenic efficacy of peripherally restricted CB-1 antagonists has been well demonstrated in rodents (4).

## Version 1 01/04/2022

Electronic publication
